# O01 ‘Matters of the heart’: navigating resistance and allergies in *Granulicatella adiacens* endocarditis

**DOI:** 10.1093/jacamr/dlaf230.001

**Published:** 2025-12-04

**Authors:** N Reidy, S Lapthorne, S Kenny, T Healy, S McLoughlin, A Doherty, C Hickey, G D Corcoran, N Mullane, M Hannan, K Doddakula, C Sadlier, J Walsh

**Affiliations:** Department of Clinical Microbiology, Cork University Hospital, Ireland; Department of Clinical Microbiology, Cork University Hospital, Ireland; Department of Clinical Microbiology, Cork University Hospital, Ireland; Department of Clinical Microbiology, Cork University Hospital, Ireland; Department of Clinical Microbiology, Cork University Hospital, Ireland; Department of Clinical Microbiology, Cork University Hospital, Ireland; Department of Clinical Microbiology, Cork University Hospital, Ireland; Department of Clinical Microbiology, Cork University Hospital, Ireland; Department of Clinical Microbiology, Cork University Hospital, Ireland; Department of Clinical Microbiology, Mater Misericordiae University Hospital, Ireland; Department of Cardiothoracic Surgery, Cork University Hospital, Ireland; Department of Infectious Diseases, Cork University Hospital, Ireland; Department of Clinical Microbiology, Cork University Hospital, Ireland

## Abstract

**Background:**

Nutritionally variant streptococci (NVS), including *Granulicatella* and *Abiotrophia* genera, are fastidious Gram-positive cocci requiring pyridoxal or thiol for growth. They are implicated in 1%–2% of infective endocarditis (IE) cases and associated with large vegetations and poorer outcomes.¹ Multiple reports describe treatment of *Granulicatella adiacens* IE with guideline-supported regimens such as penicillin, ceftriaxone, and vancomycin.^2–4^ We present a case of *G. adiacens* IE in a patient with aortic homograft, with ceftriaxone resistance and allergies to penicillin and vancomycin, and review literature on treatment of NVS IE with alternative agents outside of recognized guidelines.

**Case report:**

A 45-year-old female with Turner’s syndrome, prior bioprosthetic aortic valve replacement and aortic homograft presented with one month of malaise, dyspnoea and fever. Echocardiography revealed severe aortic regurgitation with a 9×4 mm aortic valve vegetation. Four sets of blood culture cultured *G. adiacens* on standard blood agar without pyridoxal supplementation. Empirical ceftriaxone, vancomycin and gentamicin were commenced, pending susceptibility testing. She underwent aortic valve replacement. Valve culture was negative, but 16S PCR detected *G. adiacens*. Reference laboratory susceptibility testing demonstrated ceftriaxone resistance and penicillin/vancomycin susceptibility. Amoxicillin and vancomycin were then initiated, while amoxicillin susceptibility result was awaited. The patient developed a severe cutaneous reaction with eosinophilia of 0.93×10⁹/L, meeting criteria for DRESS syndrome (drug rash with eosinophilia and systemic symptoms), with β-lactams suspected as culprits. Amoxicillin was stopped; however the rash worsened, prompting vancomycin discontinuation also. Due to fastidious growth of *G.* adiacens and delayed susceptibility results, meropenem and linezolid were trialled empirically. Thrombocytopenia subsequently developed on linezolid (nadir platelet count 114×10⁹/L), which was ceased. Ultimately, she completed 6 weeks of therapy post-surgery with ertapenem and levofloxacin, with good clinical response. MICs were 0.064 for ertapenem and 0.5 for levofloxacin—while no EUCAST breakpoints exist for *G. adiacens*, the reference laboratory suggested both may be considered for therapy . Surveillance blood cultures one month after completing therapy were negative and repeat echocardiography showed a well-seated prosthetic valve with no vegetation.

**Discussion:**

Infective endocarditis guidelines from AHA, ESC and BSAC recommend penicillin, ampicillin, or ceftriaxone with gentamicin for six weeks in penicillin-susceptible *Granulicatella* IE, or vancomycin with gentamicin in β-lactam allergy.^2,3,4^ Data on daptomycin, linezolid and fluoroquinolone use for NVS IE are limited. One study found 87% of *G. adiacens* isolates meropenem-susceptible.⁵ Fastidious growth, prolonged turnaround for susceptibilities and lack of formal EUCAST breakpoints complicated antimicrobial selection in this case. Recurrence of NVS IE is common (17%–27%),⁶ and the retained aortic homograft raised relapse risk. Long-term oral suppressive therapy was avoided due to limited options and fluoroquinolone toxicity concerns. Instead, close outpatient monitoring was instituted.

**Conclusions:**

The management of NVS endocarditis is already challenging due to its fastidious growth, more severe prognosis, high relapse rates and antibiotic resistances. This case was further complicated by severe antibiotic hypersensitivity reactions to guideline-supported antibiotic regimens. Guided by case series and MDT input, a non-guideline-supported regimen achieved successful outcomes. Publication of such cases is essential to inform future management strategies for rare and difficult infections.

## References

[1] Adam EL et al Case series of infective endocarditis caused by *Granulicatella* species. Int J f Infect Dis 2015; 31: 56–8. 10.1016/j.ijid.2014.10.023 25461651

[2] Baddour LM et al; American Heart Association Committee on Rheumatic Fever, Endocarditis, and Kawasaki Disease of the Council on Cardiovascular Disease in the Young, Council on Clinical Cardiology, Council on Cardiovascular Surgery and Anesthesia, and Stroke Council. Infective endocarditis in adults: diagnosis, antimicrobial therapy, and management of complications: a scientific statement for healthcare professionals from the American Heart Association. Circulation 2015; 132: 1435–86. 26373316 10.1161/CIR.0000000000000296

[3] Delgado V et al; ESC Scientific Document Group, 2023 ESC Guidelines for the management of endocarditis: developed by the task force on the management of endocarditis of the European Society of Cardiology (ESC) Endorsed by the European Association for Cardio-Thoracic Surgery (EACTS) and the European Association of Nuclear Medicine (EANM). Eur Heart J 2023; 44: 3948–4042. 37738322 10.1093/eurheartj/ehad625

[4] Gould FK et al Guidelines for the diagnosis and antibiotic treatment of endocarditis in adults: a report of the Working Party of the British Society for Antimicrobial Chemotherapy. J Antimicrob Chemother 2011; 67: 269–89. 22086858 10.1093/jac/dkr450

[5] Mushtaq A et al Differential antimicrobial susceptibilities of *Granulicatella adiacens* and *Abiotrophia defectiva*. Antimicrob Agents Chemother 2016; 60: 5036–9. 27216060 10.1128/AAC.00485-16 PMC4958207

[6] Fowler VG et al Endocarditis and intravascular infections, chapter 74. In: Mandell GL, Bennett JE, Dolin R, eds Mandell, Douglas and Bennett's Principles and Practice of Infectious Diseases. Vol. 1, 6th edn. Churchill Livingstone, 2005; 988–9.

